# Potential Pitfalls of the Humanized Mice in Modeling Sepsis

**DOI:** 10.1155/2018/6563454

**Published:** 2018-09-02

**Authors:** Krzysztof Laudanski, Michael Stentz, Matthew DiMeglio, William Furey, Toby Steinberg, Arpit Patel

**Affiliations:** ^1^Department of Anesthesiology and Critical Care, University of Pennsylvania, Philadelphia, PA 19104, USA; ^2^Department of Anesthesiology and Intensive Care, Emory University, Atlanta, GA 30322, USA; ^3^Philadelphia College of Osteopathic Medicine, Philadelphia, PA 19131, USA

## Abstract

Humanized mice are a state-of-the-art tool used to study several diseases, helping to close the gap between mice and human immunology. This review focuses on the potential obstacles in the analysis of immune system performance between humans and humanized mice in the context of severe acute inflammation as seen in sepsis or other critical care illnesses. The extent to which the reconstituted human immune system in mice adequately compares to the performance of the human immune system in human hosts is still an evolving question. Although certain viral and protozoan infections can be replicated in humanized mice, whether a highly complex and dynamic systemic inflammation like sepsis can be accurately represented by current humanized mouse models in a clinically translatable manner is unclear. Humanized mice are xenotransplant animals in the most general terms. Several organs (e.g., bone marrow mesenchymal cells, endothelium) cannot interact with the grafted human leukocytes effectively due to species specificity. Also the interaction between mice gut flora and the human immune system may be paradoxical. Often, grafting is performed utilizing an identical batch of stem cells in highly inbred animals which fails to account for human heterogeneity. Limiting factors include the substantial cost and restricting supply of animals. Finally, humanized mice offer an opportunity to gain knowledge of human-like conditions, requiring careful data interpretation just as in nonhumanized animals.

## 1. Introduction

Animal models are frequently employed as a precursor to clinical trials and even more broadly in research investigations. Despite their enormous contribution to the development of scientific knowledge, there is a well-funded and accepted appreciation of their limitations [1–3]. Animal and human physiology may be similar, but this is not a universal rule [4]. For example, the toll-like receptor pathway is relatively conserved in both humans and* Drosophila*, but the clinical response to ligands varies between animals and humans [5]. These differences are sufficiently profound to prevent the successful, direct translation of discoveries in mice into human clinical trials. However, the high prevalence and mortality resulting from sepsis necessitate further research in the field of critical care inflammation using animal models [2, 6].

Some of the issues hampering clinical implementation of discoveries in animal models are related to the shortcomings of the general methodology of animal experimentation [3]. Animals are often inbred and kept in sterile conditions, leading to high homogeneity. While reducing interindividual variability is helpful in pure laboratory research, the applicability of the research may be compromised by the lack of diversity typical of human patients. It is also questionable how well the environment of the animal facility resembles natural conditions. Stable temperature, rigorously controlled diet, multiple barriers to prevent infection, and limitations on animal mobility due to housing constraints are far from typical clinical environments [2, 7]. Furthermore, disease models are only an approximation of illness and may not correspond well to clinical scenarios [8]. For example, cecal ligation and puncture are a widely employed model of sepsis in rodents, but they have been criticized for not adequately reflecting typical clinical conditions and treatment [3, 9]. In particular, antibiotic and fluid management are dramatically different between lab animals and clinical settings [2, 10]. Lastly, rodent models offer only limited efficacy in testing several higher-order neurological functions such as cognition, memory, and emotional regulation which makes modeling of inflammation-induced dysfunction of central nervous system somewhat difficult.

Numerous papers have been published debating the utility of animal models [3, 4, 8, 9]. There is little doubt that animal models are essential research tools, but increased concerns have triggered a search for alternative methods to investigate important clinical questions [3, 4, 8, 11]. With the National Institutes of Health (NIH) emphasizing “translational research,” there is added impetus to look for these alternatives.

One promising approach to bridging this translational gap is the application of “humanized mice.” Here, we will review the basic understanding of their immunology and their applicability to human disease in the context of acute critical care illness. Our review discusses certain characteristics of the humanized mice model that are potentially limiting their capacity for direct translation of research findings into clinical therapy [9, 12].

## 2. Development of “the Ideal” Humanized Mice

Humanized mice can be defined simply as mice carrying human genes or tissues such as leukocytes, stem cells, organs, and tumors [12]. In this review, we will focus only on animals with a reconstituted immune system and not mice with transplanted human neoplastic tissues, human pancreatic islets, cardiomyocytes, or other biological materials. A viable humanized mouse model is contingent on the successful development of an immunodeficient host enabling the native immune system to combat the transplanted one. Consequently, the primary goal of humanized mice development is to engineer mice with increasingly deficient native immune systems to curb the rejection of the transplanted immune system [13–15]. The secondary goal is aimed at providing the grafted cells with a specific microenvironment in which they can successfully settle and thrive [16].

### 2.1. Development of Immunocompromised Host

The earliest model consisted of CB17 mice strain carrying the* Prkdc*^scid^ mutation leading to severe combined immunodeficiency (SCID) [13, 16]. The first attempt was a simple transplant of human peripheral leukocytes into mice [14]. HIV-1 was the primary impetus for the development of this model, notwithstanding issues with efficient grafting and graft stability with overrepresentation of NK cell activity. The development of nonobese diabetic- (NOD-)* scid* mice further improved the engraftment in comparison to previous immunodeficient host types [17]. The success of this subsequent model was attributed to its decreased NK cell activity and the emergence of additional defects of innate immunity, which allowed for higher levels of engraftment [10, 18]. Null mutations in the IL-2 receptor *γ* chain (*IL2rg*^null^) were particularly successful in attaining this goal. Consequently, mouse strains including IL-2 receptor *γ* chain mutations such as NOD/Shi-*scid*/*IL2rγ*^−/−^ (NOG), NOD-*Rag1*^−/−^*IL2rγ*^−/−^ (NRG), NOD-*scid/IL2rγ*^−/−^ (NSG), or BALB/c/*Rag2*^−/−^*IL2rγ*^−/−^ (BRG) were widely adapted for their best hosting capability [15, 18–20]. The deletion of *β*2-microglobulin (NOD-*said B2m*^−/−^), a critical component of MHC I, further improved engraftment compared to NOD-*SCID* mice [16]. The most unique feature of the NOD-*Rag1*^−/−^ mice was generation of the first radiation-resistant strain, though engraftment was less efficient as compared to previous strains [19, 21]. Progress continued in developing mice strains with increasingly deficient native immune systems improving graft success and stability. Subsequently, the newest types of humanized mice have satisfactory recovery of T cells, B cells, and NK cells despite some variability [22–24]. At the same time, they suffer from diminished monocyte and dendritic cell counts coupled with frequently underperforming function of their humanized leukocytes [25–27]. High variability in emergence of the human mononuclear component results from the variable expression of signal regulatory protein-*α* (SIRP-*α*) [28].

The immunocompromised host retains a significant part of the native immune system despite all aforementioned interventions. Consequently, all models are at risk of developing graft versus host disease (GVHD) since transplanted CD8^+^ cells will interact with MHC class I of the host [15, 28, 29]. Incomplete eradication of the native immune system results in the development of lymphomas whereby more advanced humanized models have had much longer lag time and a lower propensity allowing for prolonged longitudinal studies [30].

### 2.2. Evolution of Grafting

First, researchers observed that human peripheral blood mononuclear cells (PBMCs) could engraft successfully [14, 31]. While injecting mice with PBMCs resulted in the reconstitution of T cells, the high risk of graft versus host disease limited such models to short-term experiments [14]. In next step human fetal thymus and liver were implanted into the kidney capsule and CD34^+^ cells from bone marrow (BLT model) [32]. This system preserved the complex interaction between antigen presenting cells (APCs) and T cells, which is critical in the resolution of acute inflammation [8, 10, 32]. MHC restricted selection of T cells took place in this model. Furthermore, a secondary lymphatic system developed along the human mucosal immune system. In the next step, CD34^+^ stem cells were used for grafting allowing for even more complete restoration of the immune system (HIS model) [33, 34]. This model featured high graft stability and diversity, proportional to the number of cells used for grafting [33–36].

Due to a lack of species-specific cytokines and inefficient ability of host mesenchymal cells to support function of the leukocytes, an emergence of a more complete human immune system was impaired [23, 37, 38]. Lack of interlocking cytokine networks contributed to the poor regulation of leukocyte populations [39]. Nanoparticles, plasmids, and lentiviruses were tried to boost the supportive environment of mice bone marrow since optimal cytokine environment is pivotal for the emergence of a complete immune system [21, 38, 39]. Despite these efforts and several other modifications, the development of most specialized cells was hampered [40, 41]. Even more alarming was the emergence of unintended consequences of genetic manipulation aimed at boosting the production of human cytokines. For example, Billerbeck et al. found that increased expression of stem cells factor, IL-3, and GM-CSF led not only to the increased expression of monocytes but also to the significant skewness of the circulating T cells toward Fox3(+) T cells [37]. Aberration of composition of leukocyte population is one of the critical phenomena of sepsis. Consequently, an introduction of any bias may affect the validity of results and their translation into clinical practice. Currently, engineering of MIRTG mice, which can produce four human cytokines endogenously, is the most sophisticated model even among similar models [23, 40, 41]. Assessment of MIRTG mice performance in sepsis follows.

### 2.3. Additional Techniques

Ancillary techniques surrounding grafting procedures have evolved as well. Initially, all animals were subjected to irradiation to remove the native immune system [15–17, 19, 33, 34, 36, 40]. However, collateral damage to supportive structures of bone marrow and organs was often present. Humanized mice with* Rag2* mutations appeared to be less sensitive to the effects of radiation [19, 41]. Some mice strains such as those carrying the* c-kit* mutation did not need irradiation, but they have not been evaluated in sepsis studies [42]. Finally, chemical ablation can be used, but it appears to have a detrimental effect on animal survival [34].

Since murine monocytes and other indigenous phagocytic cells are responsible for poor engraftment, several targeted techniques aimed at their eradication were developed such as the use of anti-murine SIRP*α*, CD47, or Ncf1 knockout, and clodronate liposomes [42–44].

Additionally, reconstitution of the immune system was found to be more efficient and better tolerated in newborn versus adult mice especially if augmented by the injection of human grafting factors [12].

## 3. The Performance of Immune System Components in Humanized Mice

Reconstitution of the human immune system is a relatively slow process that may take up to 3 months [53]. B cells are the earliest leukocytes to reconstitute, followed by T cells [18]. B cells in humanized mice produce all classes of immunoglobulins [22, 53, 58–60]. These data should be viewed with caution since the maturation of B cells and antibody class switching from IgM to IgG are particularly inefficient as compared to the human system [61, 62]. Effective immunoglobulin class switching is critical for recovery from an acute infectious process. The lack of human IL-6 is considered one of the most important reasons for this inefficiency. Only recently, a novel model of humanized mice with active human IL-6 gene was described. Interestingly, while the immunoglobulin switch was more efficient in this model, the maturation of B cells remained impaired [63]. Evaluation of these IL-6 boosted mice should be noteworthy as IL-6 is one of the critical cytokines in the development of sepsis. Additionally, the introduction of human stem cell factor, granulocyte macrophage stimulating factor, and IL-3 into the BLT model resulted in more efficient maturation and optimized immunoglobulin production at baseline and after viral infection [38].

T cells obtained from humanized mice after reconstitution are proficient as measured by the delayed hypersensitivity reaction, but their ability to respond to antigen was suboptimal [45]. Introduction of the BLT model partially resolved this problem as human T cells rely on grafted fetal thymus for clonal selection [53]. The performance of T cells depended on several factors but was mostly mediated by stimulation via MHC class II or IL-2R [22]. Since humanized mice are xenotransplanted animals, it is unclear how a state of tolerance to different MHC antigens affects their performance. Lack of MHC-matched APCs would impair that process [25, 33, 64]. Conversely, an additional transplant of sensitized dendritic cells alleviated the problem and presented an interesting opportunity for future research [54]. Altering the cytokine environment was another way to improve the presence of APCs like dendritic cells (DCs) [33, 54, 65–67]. In critical care illnesses, like sepsis, dendritic cells emerge from circulating monocytes that stimulate optimal T cell responses [10, 32]. Recently, only one study has investigated monocyte function in sepsis using humanized mice [55]. Other T cell populations may encounter similar difficulties as well. Reconstitution of the T cells in mucosal membranes depends on the presence of* γδ* chain, but its expression was variable in humanized mice [68]. Since this subtype of T cells plays a critical role in the emergence of the tolerance and the modulation of complex T cell responses, it is unclear how the deficit will affect the evolution of the immune response in the setting of critical care illness. T cells with regulatory properties (T_reg_) are present in humanized mice, but their role seems to be conflicting in terms of function and number, and it is greatly influenced by the cytokine environment [37, 69]. It is also worth mentioning that balance between different T cell populations may be abnormal in humanized mice and was linked to a deficiency of the human cytokine network [22, 29, 37, 45, 48].

Myeloid cells are the last to reconstitute after grafting. Stem cell factor, M-CSF, GM-CSF, and IL-4 are supportive in speed and efficiency of the recovery as well as in functional maturation [23, 70]. Slow and incomplete reconstitution of the myeloid line results in the inability of T cells to mount a proficient response to antigen challenges. Enhanced recovery of the myeloid compartment was seen in MISTRG and MITRG mice [23]. MO obtained from humanized mice resemble neonatal cells in their ability to upregulate CD80/CD86, two critical factors in modulating T cell function [25]. MO from humanized animals were shown to generate a robust T cell response and cytokine production after sepsis [35, 55]. Supplementation of the humanized mice with* in vitro* generated allogeneic DCs can restore T cell responsiveness [46, 64, 65]. DCs emerge in some humanized models on their own or after Flt3 supplementation [46, 49]. Such* in vivo*-generated and antigen-sensitive DCs can trigger T cell response to a specific antigen [49].

In summary, these studies show that the function of several leukocyte populations can be restored during reconstitution of the immune system. However, the complex nature of the process, dependence on numerous interventions, and unclear functional competency of leukocytes undermine the robustness of the humanized model.

## 4. Current Studies of Humanized Mice and Sepsis

Humanized mice were used successfully to study HIV [19, 30]. Most recently, several other viral infections were successfully modeled in humanized mice including Zika and West Nile virus [59, 71]. Introduction of Epstein-Barr virus reproduced several traits of the infection with high fidelity in humanized mice [40]. The ability to replicate the trajectory of viral hepatitis, longevity, and mimicry of response to subsequent infections made humanized mice especially suitable for finding the optimal drug to cure hepatitis C [71]. The endothelial inflammation of the highly lethal dengue virus and other pathological agents causing hemorrhagic fever were investigated in humanized mice, but concerns were raised regarding the accuracy of the model [60, 72, 73]. Of primary concern was the ability of the xenotransplant to mimic vasculitis and interactions between mice endothelium and human immune system [55, 74–76]. This illustrates a typical shortcoming of humanized mice when the interaction between two organs encounters an interspecies difference that can be overcome only through further modification of the models (Table 1). Whether these modifications make the model closer to reality or more artificial remains to be ascertained.

The success of humanized mice in mimicking viral infections established high expectations for using them to study sepsis [2, 32].

Sepsis is a highly prevalent and serious condition that has a profound and prolonged impact on morbidity and mortality [6, 40, 55, 74–77]. Unsinger et al. demonstrated that humanized mice replicate several key features of the septic process, including apoptosis and exaggerated cytokine production [10]. Bone marrow suppression closely resembling the natural history of sepsis was seen as well [77]. The critical role of HMGB1, TLR4, and Notch in sepsis and apoptosis was also demonstrated using humanized mice [67, 76–78]. However, the degree to which these processes replicate the complexity of the septic response is difficult to assess fully. For example, IL-15, a critical cytokine for the development of sepsis-related apoptosis, can be studied in humanized mice only after modification of the model still resulting in production of age-dependent IL-15 [32, 39, 78]. Introduction of several human cytokines improved cell recovery but introduced artificially skewed populations [37]. It was demonstrated that extended depression in bone marrow function is mediated by methylation changes in the PU.1 gene, and retransplantation of the postsepsis surviving mice with allogeneic stem cells partially restored immune reactivity [55]. However, M-CSF production and biological activities were limited to transplanted stem cells since the mice environment did not provide indigenously produced cross-reactive cytokines [66, 70]. It becomes evident that these observations may not reflect clinical reality with so many features absent from the model.

Other humans disease models successfully mimicked in humanized mice include Toxic Shock Syndrome Toxin-1- (TSST-1-) mediated shock and staphylococcal infections [40, 76]. To date, only a few research investigations have focused on sepsis in humanized mice [10, 27, 55, 67, 76, 77]. The CLP model of sepsis is by far the most popular for studying sepsis despite several shortcomings of the CLP itself and some unique features of humanized mice undergoing sepsis.

Further studies with humanized mice tested clinical compounds for the treatment of sepsis. Wang et al. tested the potential of curcumin analogs to reverse lung injury secondary to sepsis in humanized mice [27]. Autologous stem cell transplants showed a potential to reverse some of the postseptic immune system aberrations, but clinical relevance remains to be seen [55]. In another example, antibodies to human-specific toxins were tested [79]. Finally, a researcher utilized humanized mice to test indomethacin as the modulator of the immune response in neonatal sepsis under the assumption that the functional immaturity of the grafted immune system was a good model of neonatal immunity [79, 80]. These results are guarded due to insufficient evidence as to whether immature humanized mice and neonatal immunology are in fact equivalent.

## 5. Limitations of Humanized Mice Models to Study Sepsis

Humanized mice seem to be an appealing choice to investigate the pathology and treatment of sepsis [2, 10, 32, 75]. However, interest remains relatively low. PubMed cites approximately 90 publications for which “*sepsis*” and “*humanized mice*” are keywords. Many of the authors assume that several limitations of humanized mice prevent broader implementation into mainstream septic research (Table 1). Humanized mice earned justifiable praise from several researchers. However, the inherited problems of this model have been acknowledged by a few [8, 10, 12, 29, 42, 62, 77, 81].

First, there exist fundamental differences between human and mice physiology [11]. More specifically, humanized mice exhibit several differences in the natural history of sepsis. Weight loss and mortality are greater in humanized mice than wild-type mice when short-term and long-term data are analyzed [35, 55, 67, 81]. Only the introduction of extensive measures (antibiotics, fluid resuscitation, and diet modification) resulted in animal survival exceeding a couple days [35, 55]. Prolonged studies were complicated by the emergence of GVHD and lymphomas as well as the suppressive effect of preserved components of the indigenous mice immune system [15, 29, 82]. Additionally, the recovery of the granulocyte compartment required supplementation of human G-CSF [50]. Considering that granulocytes are a pivotal defense against microbial infections, this need for supplementation is a shortcoming of humanized mice in mimicking their function and heterogeneity significantly limiting that model [2, 32]. The deficit in the granulocyte compartment may also underlie early mortality in sepsis and require a more aggressive therapeutic approach [55]. Furthermore, all leukocyte types demonstrate a sign of functional immaturity unless remedial measures are implemented [25, 31, 36, 46, 66]. The corrective modification may create an artificial condition on its own or only be partially effective [37, 38]. In the example of T_reg_ expansion in humanized cells, the imbalance may not reflect the natural history of sepsis or postseptic leukocyte population changes [37]. Other shifts in T cell population composition were reported, but virtually no study investigated population heterogeneity of monocytes, NK, and B cells in the context of the response to infection [35, 45, 55]. Finally, another limitation exists in that some of the pivotal cells of the immune system are not replaced in grafting. Particularly, microglia are not part of the humanized grafted system with potentially profound negative effects on the modeling of the central nervous system's effects from sepsis.

Second, the time after grafting is critical for modulating immune responses. A minimum of 8 weeks is required for NSG grafting but longer periods are related to the emergence of lymphoma and GVHD [28, 50, 60, 82]. The question of how to measure the age of humanized mice remains. Possible measurements include host's age, grafting time, or the age of the grafted human immune system. This important question has some biological underpinnings since the production of cytokines in response to pathogens has been reported as a variable dependent on the amount of time after grafting [78]. Since sepsis disproportionally affects the lower and upper extremes of age, younger mice are still reconstituting their immune system while older ones are at higher risk of immune system disorders; humanized mice may not be the most suitable model in age-related studies of sepsis [2, 6, 32, 83].

To improve grafting, animals are frequently irradiated. Despite wide acceptance of this process, the effect of irradiation on humanized mouse physiology and sepsis remains complex and extends beyond just the immune system [56, 84]. Certain humanized models do not require irradiation, but none of them have been evaluated in septic conditions [19, 24, 85].

Third, gut flora has a significant effect on the performance of the immune system and is increasingly perceived as one of the modifiers for sepsis trajectory [2, 32]. Humanized mice have mouse gut flora interacting with the human immune system, but significant alterations are also introduced by irradiation [41, 84]. Some attempts to establish human-specific flora in humanized animals have been tried to increase the fidelity of the models since gut immune interaction is gaining increased recognition in the pathology of sepsis [72].

Fourth, sepsis is a multiorgan disease with poorly defined etiologies. The multiple interactions between pathogen and host organs as well as between the host organs themselves are crucial for understanding the clinical trajectory [2, 32, 85]. Mesenchymal cells, the central nervous system, and endothelium are frequently quoted culprits of unfavorable outcomes in sepsis [57, 85, 86]. Several deficits in the crosstalk between the grafted human immune system and mice organs may create a translation barrier for implementation of humanized mice into sepsis research [73]. Their xenotransplant nature inherently limits their usefulness as it was already suggested in studies of hemorrhagic fever or meningitis [57, 86]. Additionally, the endothelium is critical for immune system performance, and humanized mice are deemed a less favorable model for them unless significant modification is introduced [57]. Moreover, on the level of immune system interaction between APC and effector cells is notably ineffective. Vagal nerve or subcortical structures have also been suggested as playing a role in sepsis outcomes [2, 32, 86]. Meanwhile, it is unclear how the murine nervous system interacts with the human immune system during critical care illness. Lastly, mesenchymal cells are not able to have crosstalk efficiently with the grafted human immune system unless modified [51].

These are significant limitations of humanized mice as a model of sepsis due to the xenotransplant nature of these animals. In contrast, humanized mice are robust models to study pathogen cycles or relatively simple, most likely conservative, immunological processes since these illnesses trigger very specific immune system response or have a life cycle that is independent of the immune system [40, 59, 71, 72, 82].

Fifth, one often-overlooked limitation of the humanized mouse model is the statistical approach to the grafted animals. For example, several mice can be injected with the same stem cell. It is then debatable whether these mice represent one organism/ecosystem repeated several times or if they are independent experiments. Using the same cells in highly modified NSG mice would intuitively support the former conclusion. On the other hand, the interaction of the immune system with the mouse body results in unavoidable (and perhaps desirable) stochastic variation in the characteristics of the immune system.

Finally, one cannot ignore the costly nature of the humanized mice model. The high initial cost derives from the cost of stem cells used for grafting since the efficiency of grafting is proportional to the cells used for grafting. Furthermore, the animals must be housed in exceptionally protective conditions and may require a special maintenance regimen. Often, mice production is lengthy and, in some cases, restricted by patents.

## 6. Conclusions

In conclusion, humanized mice promise a better approximation of human physiology and are often recommended as a tool to bridge the gap between rodent models and clinical scenarios. However, such a view is potentially oversimplified. Our review shows that humanized mice have several unclear biases, which may profoundly affect their ability to mimic clinically relevant scenarios of sepsis. Furthermore, the significant cost and limitations associated with this model may not justify their use. There is little doubt that humanized mice are a useful tool to study the partial mechanism of sepsis, but the complexity of this disease demands more sophisticated models whereby several complex systems may interact with each other.

## Figures and Tables

**Table 1:** Pitfalls of humanized models and the means to compensate them.

*Problem definition*	*Impact on progress in research*	*Ways to overcome limitations*	*Ref.*
**The artificial condition of housing**	Increased susceptibility of the animals to infection, decreased immunity,	Creating a more realistic environment for housing	[2, 4, 7, 12]

**Clinical relevance of septic model in general**	Separation of the research outcome from clinical reality.	Developing more clinically relevant model by introducing fluid resuscitation and antibiotics, comparisons between humanized and non-humanized animals.	[2, 8, 10]

**Homogeneity of animals**	Decrease robustness of the findings.	Increasing diversity, developing models with different strains, engaging in cross-species research, grafting with different stem cells.	

**Preservation of mice native immune system**	Incomplete or inefficient grafting, GVHD, the emergence of lymphomas	Development of more profoundly immunosuppressed hosts, eradication of the residual immune system, knock-out of SIRP-*α*.	[13, 16, 17, 19, 24, 28, 30, 34, 45–47]

**Lack of supportive human cytokine environment**	Inefficient grafting, inefficient cytokine network and immune system regulation	Supplementation of human cytokines via various means	[31, 37, 48–52]

**Poor recovery of certain leukocyte population**	Incomplete restoration of the immune system, ineffective and clinically irrelevant responses	Introduction of HIS, BLT, MITGR models, supplementation of human cytokines via genetic engineering,	[10, 16, 19, 22, 24, 25, 33, 34, 36, 44, 53]

**Immunoglobulin switching**	Inability to mimic humoral responses	Development of human IL-6 producing mice, introduction of additional cytokine modification	[38, 54]

**Functional immaturity of human leukocytes**	The inappropriate response, difficulties in translating	Supplementation of adequate cytokine environment, ex vivo cell maturation, and supplementation	[35, 51, 55]

**Poor inter-organ communication**	Difficulty mimicking complex interaction between organs in sepsis	Additional transplantation to better mimic inter-organ interaction in the autologous/allogeneic system, the introduction of human intestinal flora	[35, 54–57]
